# Influence of Nanostructuration on PbTe Alloys Synthesized by Arc-Melting

**DOI:** 10.3390/ma12223783

**Published:** 2019-11-18

**Authors:** Javier Gainza, Federico Serrano-Sánchez, Neven Biskup, Norbert Marcel Nemes, José Luis Martínez, María Teresa Fernández-Díaz, José Antonio Alonso

**Affiliations:** 1 Instituto de Ciencia de Materiales de Madrid, C.S.I.C., Cantoblanco, E-28049 Madrid, Spain; fserrano@icmm.csic.es (F.S.-S.); martinez@icmm.csic.es (J.L.M.); ja.alonso@icmm.csic.es (J.A.A.); 2 Departamento de Física de Materiales, Universidad Complutense de Madrid, E-28040 Madrid, Spain; nbiskup@pdi.ucm.es (N.B.); nmnemes@fis.ucm.es (N.M.N.); 3 Instituto Pluridisciplinar, Universidad Complutense de Madrid, E-28040 Madrid, Spain; 4 Institut Laue Langevin, BP 156X, Grenoble F-38042, France; ferndiaz@ill.fr

**Keywords:** thermoelectrics, nanostructuration, lattice thermal conductivity, lead telluride, neutron powder diffraction

## Abstract

PbTe-based alloys have the best thermoelectric properties for intermediate temperature applications (500–900 K). We report on the preparation of pristine PbTe and two doped derivatives (Pb_0.99_Sb_0.01_Te and Ag_0.05_Sb_0.05_Pb_0.9_Te, so-called LAST18) by a fast arc-melting technique, yielding nanostructured polycrystalline pellets. XRD and neutron powder diffraction (NPD) data assessed the a slight Te deficiency for PbTe, also yielding trends on the displacement factors of the 4*a* and 4*b* sites of the cubic *Fm-3m* space group. Interestingly, SEM analysis shows the conspicuous formation of layers assembled as stackings of nano-sheets, with 20–30 nm thickness. TEM analysis shows intra-sheet nanostructuration on the 50 nm scale in the form of polycrystalline grains. Large numbers of grain boundaries are created by this nanostructuration and this may contribute to reduce the thermal conductivity to a record-low value of 1.6 Wm^−1^K^−1^ at room temperature. In LAST18, a positive Seebeck coefficient up to 600 μV K^−1^ at 450 K was observed, contributing further towards improving potential thermoelectric efficiency.

## 1. Introduction

Thermoelectric materials are relevant for a world relying on clean and sustainable energy sources. Thermoelectrics can directly convert heat into electrical energy, and their efficiency is evaluated by the figure of merit $ZT=S^{2}\sigma T/\kappa$, where S is the Seebeck coefficient, σ is the electrical conductivity, T is the absolute temperature, and κ is the total thermal conductivity, which contains the sum of the lattice $\left(\kappa_{l}\right)$ and electronic contributions $\left(\kappa_{e}\right)$ [1].

Several approaches have been developed to boost the thermoelectric performance in different ways, such as band engineering [2,3] or hierarchical architectures [4], leading to highly competitive ZT values >1.5 [5,6,7,8]. Among all these strategies, nanostructuration is a key role to bear in mind, because it is a useful tool to effectively reduce the thermal conductivity [4,9,10,11], and it has already been used in different chalcogenide-type compounds [12,13].

In recent years, tellurium-based compounds are among the most efficient thermoelectric materials, for instance, Bi_2_Te_3_, is widely used in near room temperature applications [1,14], or GeTe and its alloys [15,16,17,18,19], rare-earth tellurides [20,21,22,23,24], or PbTe [25,26,27,28,29]. PbTe-based thermoelectrics are the best performing materials in the middle-temperature range of 500 to 900 K [1,3,4,30,31], so they are one of the best options available for harvesting wasted thermal energy. In particular, Ag and Sb doping in the so-called LAST-type compounds, induce an excellent performance [30,32,33]. To further improve PbTe thermoelectric properties, it is important to understand the role that distinct defects may play on the lattice thermal conductivity [28], and their effects in the density of states near the bottom of the conduction band and the top of the valence band [25,29,34,35], as well as broadening our knowledge on their crystalline structures.

The first achievement of very low lattice thermal conductivities in these tellurides using nanoscale features consisted of designing superlattice structures grown by molecular beam epitaxy (MBE) [36] and chemical vapor deposition (CVD) [37]. As for other techniques, solvothermal routes have been used for nanostructuring to fabricate PbTe nanoboxes [38]. However, expensive and complex synthesis procedures are necessary to grow superlattices or produce nanoboxes, which make them impractical for real-world applications and devices [39]. For this reason, it is worth looking for alternative methods that lead to nanostructured and mechanically robust materials.

The effect of Sb or Ag addition in the structure of lead telluride has been studied before, concluding that the Sb-doping achieves a better thermoelectric performance [40,41], so we have synthesized this composition to be able to compare both pristine and doped samples. In the same way, Ag_0.05_Sb_0.05_Pb_0.9_Te (LAST18) was proposed several years ago as an efficient thermoelectric material above room temperature [30], and it has been extensively studied since then [42,43,44,45].

We have established arc-melting as a direct procedure to synthesize highly nanostructured Bi_2_Te_3_ [46,47] and SnSe [48] chalcogenides in short reaction times, obtaining hard pellets that could be directly used into devices. In this way, we avoid the use of sintering techniques, such as spark plasma sintering (SPS) or hot-pressing method, making the process more straightforward and easily scalable. In this work, we describe the preparation, with the same straightforward arc-melting method, of cubic PbTe and PbTe(:Sb, Ag) specimens showing a conspicuous laminar nanostructure, responsible for a significant reduction of the thermal conductivity. The samples have been structurally studied using X-ray diffraction (XRD) and neutron powder diffraction (NPD), as well as scanning electron microscopy (SEM), transmission electron microscopy (TEM) and the three main thermoelectric properties (Seebeck coefficient, resistivity, and thermal conductivity) that were measured as a function of temperature. 

## 2. Materials and Methods

The pristine PbTe compound and derivative Pb_0.99_Sb_0.01_Te and Ag_0.05_Sb_0.05_Pb_0.90_Te (LAST18) alloys were synthesized in an Edmund Buhler MAM-1 mini-arc furnace (Bodelshausen, Germany), using direct arc melting in a water-cooled copper crucible, with a tungsten electrode under purified argon atmosphere. The starting materials were pure elements of Pb (99.9%, Cerac, Milwaukee, WI, USA), Te (99.99%, Alfa Aesar, Kandel, Germany), Ag (99.99%, Cerac, Milwaukee, WI, USA), and Sb (99.5%, Alfa Aesar), which were weighted and mixed according to the stoichiometric ratio. Part of the resulting ingots was ground to powder for structural characterization, and the remaining part was pressed in a Retsch Pellet Press PP25 (Haan, Germany) under an isostatic pressure of 10 MPa, and then cut with a diamond saw in bar-shape to perform transport measurements. All thermoelectric properties were measured perpendicular to the pellet pressing direction, using a Physical Properties Measurement System (PPMS) by Quantum Design (San Diego, CA, USA) in the residual vacuum of He atmosphere, under a pressure of 0.1 MPa in the temperature range of 2 to 390 K. In complement, high-temperature measurements were performed on the cold-pressed sample in a homemade apparatus, along the direction of the pellet pressing [49]. The Hall coefficient was measured using the four-probe resistivity option of the PPMS in delta-mode with a DC current of I = 5 mA as a function of the magnetic field up to ±8 T. The density of the cold-pressed pellet was ~96% of the theoretical crystallographic density.

Phase characterization was carried out for the pulverized sample using X-Ray diffraction (XRD) on a Bruker-AXS D8 diffractometer (Karlsruhe, Germany, 40 kV, 30 mA), run by DIFFRACTPLUS software (version 2.5.0, Bruker-AXS, Karlsruhe, Germany), in Bragg–Brentano reflection geometry with Cu Kα radiation (λ = 1.5418 Å). Moreover, NPD was used to characterize the crystal structure concerning possible atomic vacancies and displacement factors. High-resolution patterns were collected in the D2B diffractometer at the Institut Laue-Langevin, Grenoble, France, in the high-flux configuration with a neutron wavelength λ = 1.549 Å at 298 K (RT) for PbTe. Typically, 2 g of the samples were measured in a vanadium can. Diffraction data were analyzed using the Rietveld method employing the FULLPROF program (version Sept 2018, Grenoble, France). The line shape of the diffraction peaks was modeled by a pseudo-Voigt function. The following parameters were refined in the final run for all the atoms: scale factor, zero shift, background points, pseudo-Voigt corrected for asymmetry parameters, half-width, unit-cell parameters, and isotropic displacement factors. Occupancy factors for Te atoms were also refined from NPD data. The coherent scattering lengths of Pb, Te, Ag, and Sb were 9.405, 5.80, 5.992, and 5.57 fm, respectively. High-resolution FE-SEM images were collected in an FEI-Nova NanoSEM 230 microscope (Denton, Texas, TX, USA). 

Transmission electron microscopy (TEM) and scanning transmission electron microscopy (STEM) were carried out in a JEOL ARM 200 electron microscope (Peabody, MO, USA) with the aberration corrector enabling the spatial resolution of 0.8 Å when operated at 200 kV. The microscope is equipped with a Gatan Quantum electron energy loss spectrometer (EELS, Pleasanton, CA, USA).

## 3. Results and Discussion

### 3.1. Crystal Structure

**Figure 1:**
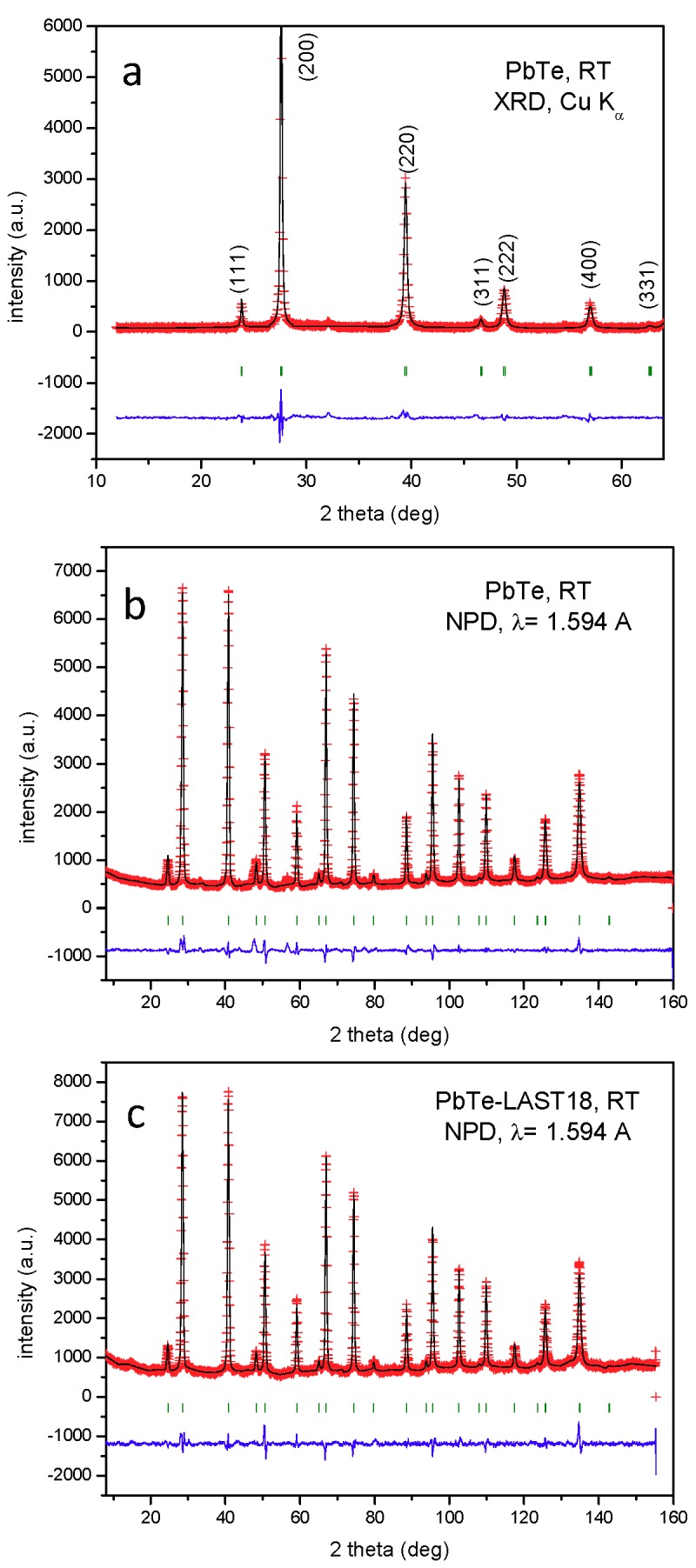
Observed (crosses), calculated (full line), and difference (at the bottom) profiles for (**a**) XRD pattern for as-grown PbTe, Rietveld-refined in the space group *Fm-3m*, (**b**) neutron powder diffraction (NPD) pattern for PbTe and (**c**) NPD patterns for PbTe-LAST18, at RT.

Figure 1 illustrates the Rietveld-refined XRD pattern of PbTe prepared by arc melting after carefully grinding the as-grown ingot. The diagram corresponds to a single-phase, well-crystallized NaCl-like structure with cubic symmetry, defined in the face-centered space group *Fm-3m*, with unit-cell parameter **a** = 6.4595(1) Å. The unit-cell size is substantially identical to that described in the literature, e.g., 6.46 Å [50]. Similar diagrams were obtained for Sb-doped and LAST18 alloys. 

Neutron powder diffraction (NPD) is essential to obtain accurate structural details of PbTe and PbTe-LAST-18. Neutrons sample a much wider range of the reciprocal space. The displacement factors can be determined precisely thanks to the lack of form factors. Furthermore, a strong aspect of this study, using NPD, is the precise characterization of any possible off-stoichiometry within the relevant crystalline phase. Losses due to evaporation are an important issue for many thermoelectrics, and NPD can keep track of the consequent stoichiometry changes, refining the occupation factor of each atom position. Despite the possible evaporation of the various elements during arc-melting, NPD showed practically the same ratio as the weighed elements before the synthesis, as described below. Technical advantages of NPD over XRD include the bulk (larger sample quantity) analysis, and the minimization of preferred orientation effects by the packing of the ground crystals in vanadium cylinders, and the rotation of the sample holder during the experiments.

The crystal structure of PbTe was refined in the NaCl-type, defined in the cubic *Fm-3m* space group [50] from NPD at RT, with Pb and Te atoms located at 4*a* (0,0,0) and 4*b* (1/2,1/2,1/2) sites, respectively. Table 1 includes the atomic parameters and displacements factors. The occupancy factors of Pb and Te could be refined and a slight Te deficiency was detected within the standard deviations (f_occ_ = 0.986(5), see Table 1). Regarding the PbTe-LAST18 specimen, we proposed a model corresponding to the stoichiometry Ag_0.05_Sb_0.05_Pb_0.90_Te (equivalent to AgPb_18_SbTe_20_) where Ag and Sb occupy at random the 4*a* sites together with Pb. This model provided good agreement factors (Table 1 and Figure 1c), observing a slight Te deficiency, only two standard deviations away from the full stoichiometry. This fact additionally assessed that Ag and Sb are not located at Te sites since both elements exhibit higher scattering lengths than Te. Remarkably, the Pb displacement factors are consistently large (≈1.8 Å^2^), and almost double than those of Te atoms. The smearing of the nuclear scattering density of Pb may be related to the presence of the lone electron pair attributable to Pb^2+^ ions (in an ionic model for lead telluride), which for the very symmetric unit cell is certainly located at random in subsequent PbTe_6_ octahedra. This implies, in each case, a displacement of the Pb atoms and the Pb-Te chemical bonds opposite to the location of the lone pair lobe. Globally, this accounts for the disorder or smearing of the scattering density, which translates into an enhanced displacement factor. 

Figure 1b,c show good agreements between observed and calculated NPD profiles for PbTe and LAST18, respectively, with correspondingly good discrepancy factors (R_Bragg_ = 2.61 and 2.06%, respectively). 

**Figure 2:**
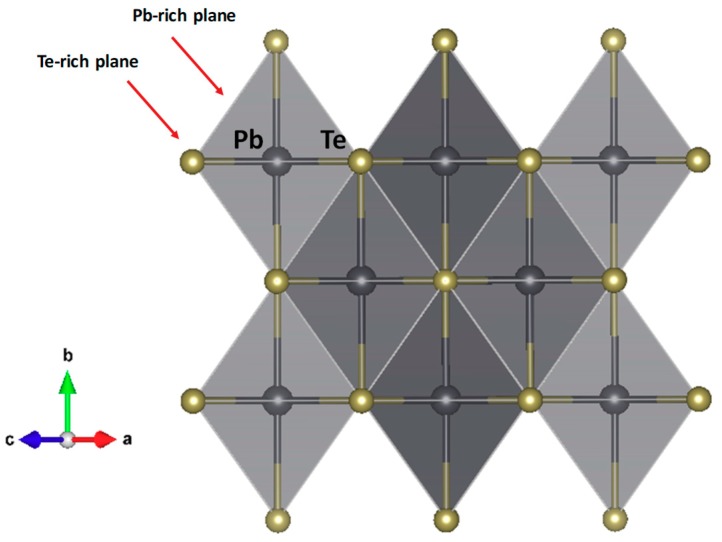
Crystal structure of PbTe along [110] directions, highlighting Te-rich planes alternating with Pb-rich planes.

Figure 2 illustrates the crystal structure of pristine PbTe, consisting of a simple NaCl arrangement of both elements in the cubic unit cell. Given the spatial positions occupied by both Pb and Te, only isotropic displacement factors can be defined. Lead atoms are coordinated to six Te atoms and vice versa. The structure contains Pb-rich and Te-rich planes along the [110] crystallographic directions, which may have important repercussions on the physical properties, providing with preferred directions for the cleavage and nanostructuration yielding layered structures, thus hindering the thermal and electronic transport across the layers, as described below.

### 3.2. Nanostructuration

**Figure 3:**
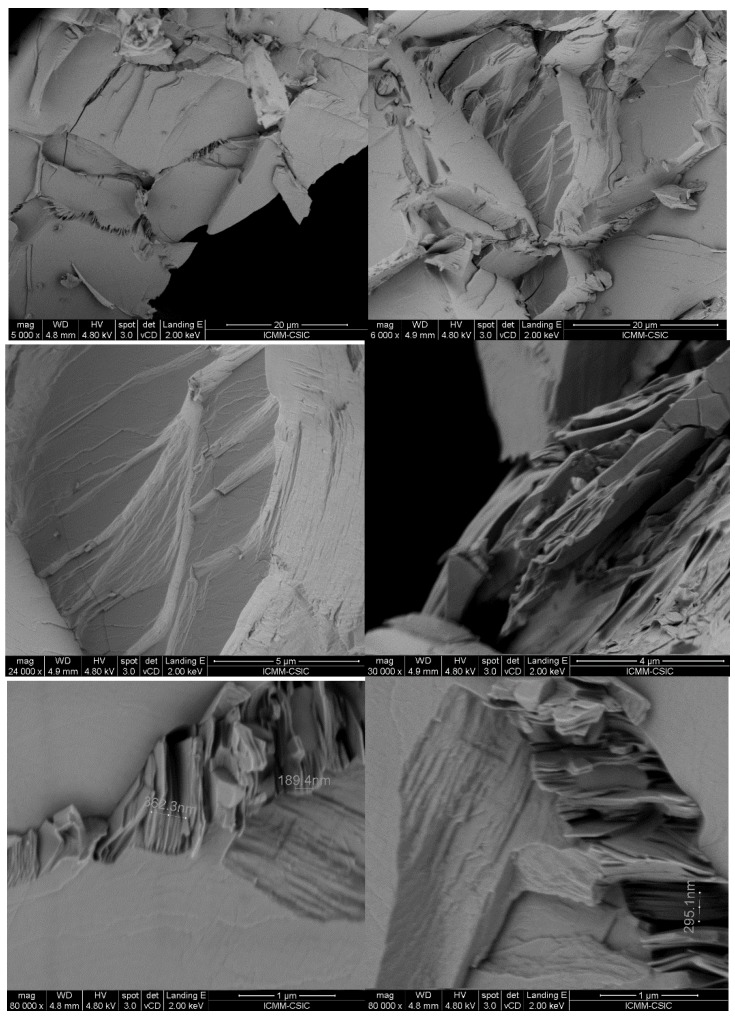
FE-SEM images displaying the superficial morphology of PbTe. A conspicuous laminar nanostructuration is achieved with the arc melting technique.

Figure 3 displays the morphology of the as-grown PbTe pellets imaged with several magnifications (from 5000× to 80,000×) by field-effect-scanning electron microscopy (FE-SEM). The as-grown ingots are formed of parallel stacked nanosized sheets, also showing growing steps, as a conspicuous feature in all the examined specimens obtained by arc-melting, from various regions of various samples. Despite the 3D-type crystal structure exhibited by these materials, a strong cleavage effect is apparent, as a consequence of weak bonding directions occurring within the crystal structure, as commented above. The approximate thickness of individual sheets can be estimated in the 20 to 30 nm range. We consider that this lamellar nanostructuring is present throughout the volume of the material, since the images of Figure 3, and many similar, were taken from different parts of the broken-up ingot. In fact, according to [51], a faster cooling rate provides more homogeneous PbTe and LAST samples. Indeed, arc-melting quenches the samples from the molten state to room temperature at extremely high cooling rates estimated to be several hundreds of degrees per second.

**Figure 4:**
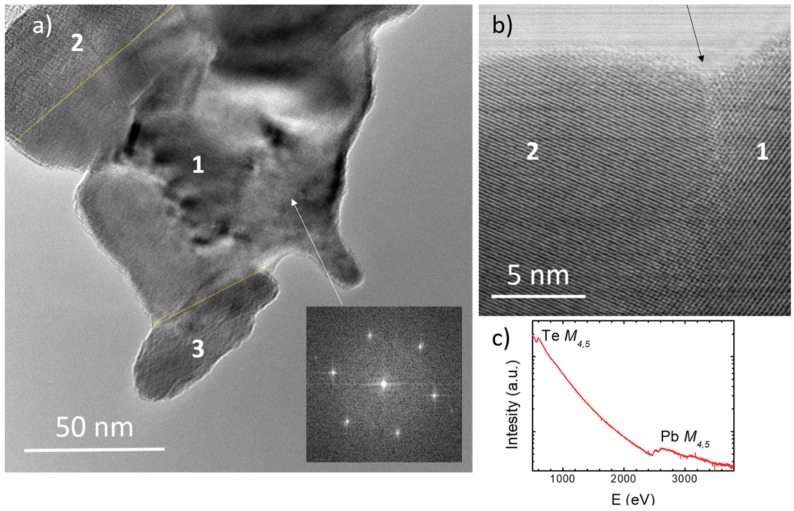
**(a)** TEM image of a conglomerate consisting of three crystalline grains. The boundaries of different grains are marked by yellow lines. The grain orientations are checked by spatially resolved diffraction patterns, the grain 1 being oriented along the [111] zone axis (inset). (**b**) High magnification image of the boundary between the grains 1 and 2. (**c**) Electron energy loss spectrometer (EELS) spectrum of the conglomerate in this figure. Only Te *M_4,5_* and Pb *M_4,5_* edges are detected.

We have examined the crystalline powder of our PbTe material with transmission electron microscopy (TEM). Figure 4a shows the TEM image of a submicron-size conglomerate. The electron energy loss spectrum (EELS, Figure 4c) shows both Te and Pb M_4,5_ absorption edges and yields 50:50 relative concentrations of both atoms. No impurities are detected by EELS, demonstrating the high purity of our material (Figure 4c). The central part of the conglomerate in Figure 4a is a single crystalline grain, as can be seen by examining diffraction patterns in different areas (inset). However, even on the scale of this image (50–100 nm), grains with different orientations can be seen: two such crystalline grain-boundaries are indicated by yellow dashed lines, and the different grains are labeled from 1 to 3. In the high magnification STEM ABF image (Figure 4b), one can see more closely one of these grain boundaries (marked by the arrow). One can conclude that our PbTe material is polycrystalline, even at a scale of 50 nm.

This extremely small size of crystalline order may contribute to the observed low thermal conductivity, due to increased phonon scattering on a larger density of grain boundaries provided by the morphology of many nanosized sheets, produced by arc-melting. 

The morphology of the PbTe based materials prepared by arc-melting is quite complex. Primarily, there are interlayer boundaries at the surfaces between neighboring nanosized sheets, which are clearly shown in the SEM images of Figure 3. Furthermore, as the high-resolution TEM images along the [111] axis shown in Figure 4 demonstrate, each nanometric layer is made of polycrystalline material that consists of single crystalline grains of 50 nm characteristic size. Thus, there are two types of nanostructuring present, both on the 20–50 nm scale. This morphology has not been reported before in PbTe specimens prepared by standard procedures (ball milling, reactions in sealed quartz capsules, etc.). Note that we use the term “grain boundary” quite generally in this study to refer to both the nanosheet-to-nanosheet interfaces (Figure 3), as well as the intra-sheet boundaries between single-crystalline grains (Figure 4b). 

### 3.3. Transport Properties

**Figure 5:**
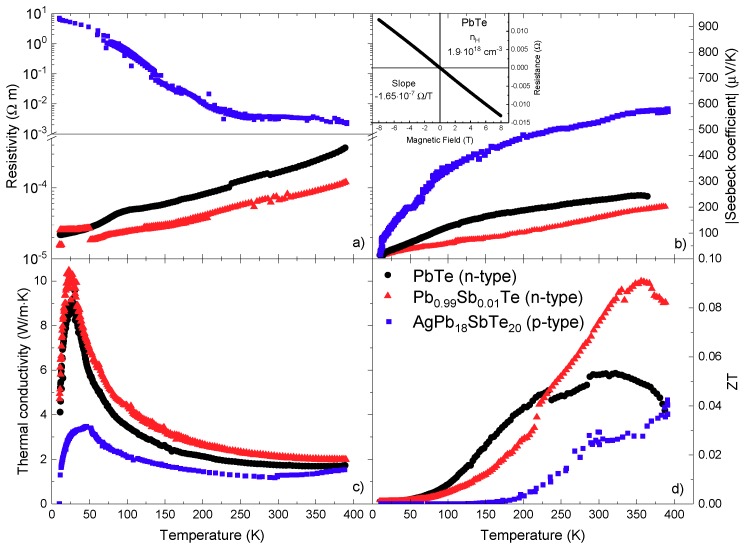
(**a**) Resistivity, (**b**) Seebeck coefficient, (**c**) thermal conductivity, and (**d**) figure of merit versus temperature for PbTe, Pb_0.99_Sb_0.01_Te, and LAST18 prepared by arc melting. The inset in (**c**) shows the Hall Effect measurement of the pristine PbTe.

**Figure 6:**
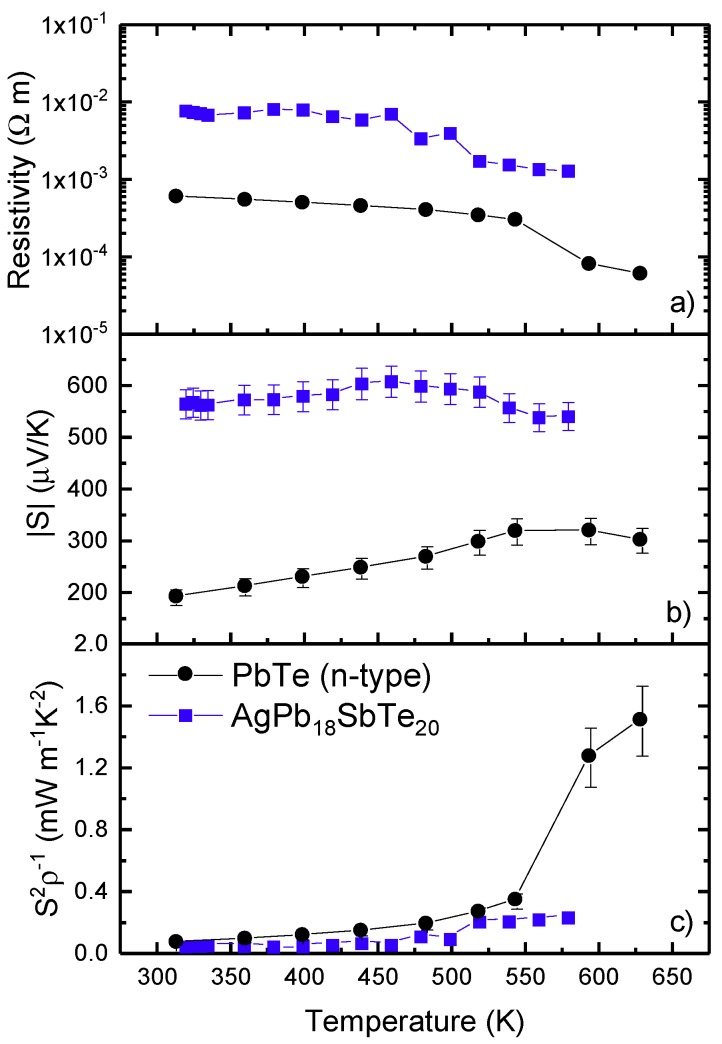
(**a**) Resistivity, (**b**) Seebeck coefficient, and (**c**) power factor above room temperature for PbTe and AgPb_18_SbTe_20_ prepared by arc melting.

The electrical resistivity and the Seebeck coefficient for PbTe, Pb_0.99_Sb_0.01_Te, and AgPb_19_SbTe_20_ are displayed in Figure 5; the corresponding curves for LAST18 data at mid-temperature are displayed in Figure 6. The resistivity of the pristine PbTe and Pb_0.99_Sb_0.01_Te increased with temperature in the 2-390 K range, as expected for metallic compounds; that of the Sb-doped sample was slightly lower, and the difference increased with temperature. For instance, at 300 K PbTe exhibited a resistivity of $1.6\times 10^{-4}\;\Omega \cdot m$ at 300 K, whereas for the Sb-doped compound it was $6.5\times 10^{-5}\;\Omega \cdot m$. It seems that the Sb doping increased the electron concentration, raising the electrical conductivity while the Seebeck coefficient (Figure 5b) was reduced (in absolute value). This behavior has been already reported in Sb-doped PbTe prepared by a melting procedure in vacuum sealed-tubes [41,52].

The behavior of the Seebeck coefficient from 10 K up to 390 K displayed in Figure 5b is similar to those reported elsewhere [2,25,53,54], where the thermopower is slowly but continuously rising up to higher temperatures. At room temperature, the Seebeck coefficient of PbTe is reported to be 150 μV/K [53], or even below that value [26], like the heavy hole-doped PbTe, which shows a Seebeck coefficient around 50 μV/K [54]. Compared with these data, the Seebeck coefficients of our samples were higher (in absolute value), reaching −220 μV/K at RT, which is also related to the higher resistivity we found, following the well-known Pisarenko relationship. The antimony doping in this sample did not alter the fact that the electrons are the majority carriers, yielding an n-type material, in accordance with the results for other Sb doping levels [40].

At room temperature, the Seebeck coefficient should be positive and then switch to a negative value with increasing temperature [55,56]. We think that the obtained behavior of the Seebeck coefficient for pristine PbTe could occur due to slight evaporation of Te during synthesis. We can identify the tiny reflection on XRD at ~32° as Pb metal. Therefore, the material could probably have a slight deviation from stoichiometry, as shown from NPD data, and also contain precipitates of Pb. That is likely the reason why the PbTe material shows n-type behavior at 300 K. The room temperature Hall Effect measurements (inset of Figure 5c) confirmed the n-type behavior of our pristine PbTe, showing a carrier concentration of $\sim 1.9\times 10^{18}\;{\mathrm{cm}}^{-3}$, similar to other reported results [40], but a bit lower than the optimum carrier concentration for n-type lead telluride, which stands between $4-40\times 10^{18}\;{\mathrm{cm}}^{-3}$ [35,57]. Considering a single parabolic band approximation and a scattering of charge carriers dominated by acoustic phonons [58], we calculated an effective mass of 0.19 m_e_, which agrees with similar reported data for n-type lead telluride, with effective masses slightly above 0.20 m_e_ [35]. 

From carrier density and resistivity, we can calculate the mobility of the electrons in PbTe at room temperature. This calculation showed a mobility of 205 cm^2^/V·s, which was relatively high, but it is still far from the best results obtained for this composition. For example, Dow et al. reported a mobility of 2955 cm^2^/V·s for a carrier density of $2.67\times 10^{18\;}{\mathrm{cm}}^{-3}$ [40], and Pei et al. found a mobility above 2000 cm^2^/V·s for a carrier concentration of $4.3\times 10^{18}\;{\mathrm{cm}}^{-3}$ [35].

Figure 5c displays temperature-dependent total thermal conductivity. It shows the expected Umklapp maximum at 30 K and then a monotonous decrease to a minimum value of 1.6 W m^−1^ K^−1^ at room temperature for pristine PbTe. For the Sb-doped specimen, a slightly higher value was observed. Because of its high resistivity, the electronic contribution to the total thermal conductivity was negligible, so we can consider the lattice and the total thermal conductivity nearly equal. In contrast, other authors reported measurements of the thermal conductivity around 2 W m^−1^ K^−1^ [35,54], for PbTe and some different alloys [53,59].

The observed reduction of the thermal conductivity with respect to the literature values could be related to the nanostructuration that was observed in the FE-SEM and the TEM images. The underlying reason for this decrease is likely the strong phonon scattering happening at the grain boundaries, associated with the sheet-type nanostructuration, which is of great importance when considering the formation of nanostructures to obtain higher figures of merit [60].

This thermal conductivity can be a good starting point to improve the thermoelectric performance of nanostructured arc-melted PbTe. It has been demonstrated that PbTe alloys, such as PbTe:Na [54], Pb_1−x_La_x_Te [35], Pb_1−x_Mn_x_Te [26], PbTe-MgTe [25] or PbTe-SrTe [59], can reach a high thermoelectric performance through the improvement in their power factor. Bearing this in mind, with an adequate doping element, a nanostructured arc-melted PbTe with a great thermoelectric performance could be produced using this fast and straightforward method.

The figure of merit of both PbTe and Pb_0.99_Sb_0.01_Te is shown in Figure 5d. The antimony doping enhanced the thermoelectric performance of PbTe. Pb_0.99_Sb_0.01_Te had a higher figure of merit than that of the pristine compound at room temperature, reaching almost 0.1. This value is similar to others reported for PbTe and PbTe-based compounds at this temperature [10,28,50,52,53]. Pei et al. [35] found a ZT of 0.3 at room temperature for PbTe for a slightly higher carrier density of $4.3\times 10^{18}\;{\mathrm{cm}}^{-3}$, the difference with this work is mainly the high mobility they achieve in their samples, reaching a mobility of around 2000 cm^2^/V·s at room temperature.

The behavior of the resistivity and the Seebeck coefficient for PbTe and LAST18 (AgPb_18_SbTe_20_) above room temperature are displayed in Figure 6a,b. The resistivity of the LAST compound decreased with temperature from 300 K up to 550 K, being slightly above, but with similar behavior, than those also found by other authors [32,33]. In the case of PbTe, its resistivity stood below $1\times 10^{-3}\;\Omega \cdot m$ from room temperature up to 550 K. For pristine PbTe, the Seebeck coefficient continued increasing (in absolute value) with temperature, with the same behavior as shown below 300 K. However, for the LAST compound, the Seebeck coefficient was positive, reaching a maximum of 620 μVK^−1^ at 480 K, and 540 μVK^−1^ at 580 K, which is significantly larger than other thermoelectric factors reported elsewhere [30,32,33], both p and n-type. There have been other attempts to substitute silver by isoelectronic copper in this composition using different synthesis routes [61], yielding similar transport properties than that of LAST-18. However, the resulting thermopower is almost 50% lower, especially at higher temperatures, which results in a lower figure of merit compared with the AgPb_18_SbTe_20_ composition.

The power factor for PbTe and LAST18 is shown in Figure 6c, calculated as S^2^/ρ using the experimental values of the resistivity and the Seebeck coefficient. Beyond 500 K, the power factor increased, reaching 0.33 and 0.23 mW m^−1^ K^−2^ for the pristine PbTe and LAST compound, respectively, indicating promising properties in this temperature range, which is where these PbTe-type compounds present their best transport properties [28,35,54]. Moreover, according to literature, a minimum deviation from the stoichiometric composition can significantly alter the thermoelectric properties [32,33], so the thermoelectric performance of LAST material could be improved, modifying the silver and lead quantity. For a more thorough comparison, data of PbTe prepared by different methods are displayed in Table 2.

Usually, the thermal conductivity of these lead telluride materials shows a monotonous decrease beyond room temperature. This allows us to calculate a lower limit for the figure of merit of this material, using the thermal conductivity at room temperature (1.6 W/mK and 1.28 W/mK for PbTe and LAST, respectively) as well as the power factor for each composition. We obtained a figure of merit of ZT~0.59 at 630 K for PbTe, quite higher than other reported data for pristine PbTe [55,56], but, of course, far from the highest figures of merit reached by other doped lead tellurides. In the case of the LAST compound, the calculated figure of merit was around ZT~0.1 at 580 K, quite lower compared with the best results reported for LAST compounds [30,32].

PbTe prepared by arc-melting shows the lowest thermal conductivity and the highest Seebeck coefficient at room temperature, although its resistivity is significantly higher compared to other synthesis routes. However, the resistivity and Seebeck coefficient can be balanced with adequate doping to enhance the general thermoelectric performance. In conjunction with the short synthesis time of arc-melting, this could result in lead telluride derivatives competitive with those synthesized by other methods.

## 4. Conclusions

Three thermoelectric materials were prepared by a straightforward arc-melting technique: pristine PbTe, Pb_0.99_Sb_0.01_Te, and Ag_0.05_Sb_0.05_Pb_0.9_Te (LAST18). All show highly nanostructured morphology with improved thermal transport properties. A structural NPD study accurately determined the displacement factors at the NaCl-type structure and assessed the almost full stoichiometry of the specimens, with a slight Te deficiency for PbTe. The trend to cleave and to form nanostructured materials, despite the 3D crystal structure, is observed by FE-SEM. TEM imaging revealed intra-sheet nanostructuration on a 50 nm scale. This nanostructuring affects the physical properties of the material, enhancing the phonon scattering and yielding a reduced thermal conductivity with respect to literature values, on samples prepared by alternative procedures. The as-grown robust pellets are suitable to be manipulated and used in thermoelectric devices, and their physical properties could be improved even more with proper doping elements.

## Figures and Tables

**Table 1:** Structural parameters of pristine PbTe and LAST-18 (AgPb_18_SbTe_20_) from NPD data at 298 K, with λ = 1.594 Å.

	PbTe	LAST-18
a(Å)	6.4595(1)	6.4590(1)
V(Å^3^)	269.522(8)	269.460(9)
*Pb,Ag,Sb 4a(0,0,0)*		
f_occ_ Pb/Ag/Sb	1.00/0.0/0.0	0.90/0.05/0.05
B(Å^2^)	1.80(3)	1.75(4)
*Te 4b (1/2,1/2,1/2)*		
f_occ_ Te	0.985(6)	0.96(1)
B(Å^2^)	1.19(4)	1.13(5)
*Agreement factors*		
R_p_ (%)	3.07	3.66
R_wp_ (%)	4.67	4.95
R_Bragg_ (%)	2.61	2.06
χ^2^	8.22	3.04
*Distances (Å)*		
Pb-Te (x6)	3.230(1)	3.229(1)

**Table 2:** Comparison of lead telluride thermoelectric properties obtained by different synthesis processes. All data are measured at 300 K.

	Thermal Conductivity (W/m^−1^K^−1^)	Seebeck Coefficient (μV/K)	Resistivity (Ω m)	Synthesis Method	Synthesis Time	Density	Reference
**PbTeM** **(This Work)** **(n = 1.9 × 10^18^)**	1.6	−215	$1\times 10^{-4}$	Arc-melting + cold pressing	~1 h	~96%	-
**PbTe**	2.25 (κ_lattice_)	-	-	Quartz tubes (Liquid matrix encapsulation)	Several hours	-	[13]
	4.25	30	$4.2\times 10^{-6}$	Quartz tubes + SPS	~60 h	-	[62]
	3.8	60	$4\times 10^{-6}$	Graphite coated quartz tubes	~62 h	-	[10]
**PbTe** **(n = 4.3 × 10^18^)**	2.1	−170	$1.2\times 10^{-5}$	Graphite coated quartz tubes + Hot pressing	8 h + 3 days of annealing	>98%	[35]
**PbTe** **(n = 9.4 × 10^19^)**	5.5	−40	$1\times 10^{-6}$			>98%	
**PbTe Nanocrystals**	1.85	150	$8\times 10^{-3}$	Low temperature route (353 K) in a micellar medium + SPS	~3.5 h	~ 98.5%	[63]
